# Maturation of Pseudo-Nucleus Compartment in *P. aeruginosa,* Infected with Giant phiKZ Phage

**DOI:** 10.3390/v12101197

**Published:** 2020-10-21

**Authors:** Yana A. Danilova, Viktoriia V. Belousova, Andrey V. Moiseenko, Innokentii E. Vishnyakov, Maria V. Yakunina, Olga S. Sokolova

**Affiliations:** 1 Biology Department, Moscow Lomonosov University, 119234 Moscow, Russia; klein.ya@yandex.ru (Y.A.D.); postmoiseenko@gmail.com (A.V.M.); 2 Research Center of Nanobiotechnologies, Peter the Great St. Petersburg Polytechnic University, 195251 St. Petersburg, Russia; vikabelva@gmail.com (V.V.B.); innvish@gmail.com (I.E.V.); yakuninam@gmail.com (M.V.Y.); 3 Semenov Institute of Chemical Physics, Russian Academy of Sciences, 117977 Moscow, Russia; 4 Institute of Cytology, Russian Academy of Sciences, 194064 St. Petersburg, Russia; 5 Laboratory of Evolution of Sensory Organs, Sechenov Institute of Evolutionary Physiology and Biochemistry Russian Academy of Sciences, 194223 St. Petersburg, Russia; 6 Biology Department, Shenzhen MSU-BIT University, Shenzhen 518172, China

**Keywords:** giant phage, phiKZ, *Pseudomonas aeruginosa*, nucleoid, pseudo-nucleus, analytical electron microscopy, electron tomography, fluorescent *in situ* hybridization, stress response

## Abstract

The giant phiKZ phage infection induces the appearance of a pseudo-nucleus inside the bacterial cytoplasm. Here, we used RT-PCR, fluorescent *in situ* hybridization (FISH), electron tomography, and analytical electron microscopy to study the morphology of this unique nucleus-like shell and to demonstrate the distribution of phiKZ and bacterial DNA in infected *Pseudomonas aeruginosa* cells. The maturation of the pseudo-nucleus was traced in short intervals for 40 min after infection and revealed the continuous spatial separation of the phage and host DNA. Immediately after ejection, phage DNA was located inside the newly-identified round compartments; at a later infection stage, it was replicated inside the pseudo-nucleus; in the mature pseudo-nucleus, a saturated internal network of filaments was observed. This network consisted of DNA bundles in complex with DNA-binding proteins. On the other hand, the bacterial nucleoid underwent significant rearrangements during phage infection, yet the host DNA did not completely degrade until at least 40 min after phage application. Energy dispersive x-ray spectroscopy (EDX) analysis revealed that, during the infection, the sulfur content in the bacterial cytoplasm increased, which suggests an increase of methionine-rich DNA-binding protein synthesis, whose role is to protect the bacterial DNA from stress caused by infection.

## 1. Introduction

Giant phiKZ-like bacteriophages of the Myoviridae family, which include: *Pseudomonas aeruginosa* phiKZ, EL, OBP and *Pseudomonas chlororaphis* 201phi2-1, possess circular genetic maps [1,2] packaged inside the capsid according to the head-full mechanism. This means that, in the course of the DNA packaging, the entire interior space of the head is filled with DNA. Sequencing of the giant phage genome revealed that the genome encodes not only the structural proteins of the phage capsid and tail but also RNA polymerases [3,4], chaperonins [1,5], and proteins of the inner body [6]. The inner body is the internal proteinaceous structure covered with genomic DNA [7]. It was discovered to be specific for giant phages [8,9]. The function of the inner body is to support the DNA inside the capsid, to participate in the genome ejection to the bacterial cell, and to form the depo of phage proteins [6]. During the ejection of DNA into the host cell, certain proteins are co-injected to build the machinery necessary for the transcription of early genes [6]. For a long time now, phage therapy [10,11] has been employed for the effective treatment of bacterial infections. Phage treatment has shown to be far more successful in comparison with antibiotic monotherapy [12].

Bacteria have developed various defense mechanisms against bacteriophage infections. These include: blocking of phage receptors, production of an extracellular matrix and the production of competitive inhibitors to prevent phage absorption; superinfection exclusion (Sie); destroying foreign DNA with the Restriction-Modification system [13,14,15]; degradation of phage genetic material and the buildup of inheritable DNA-encoded immunity ensured by clustered regularly interspaced short palindromic repeats (CRISPR), and CRISPR-associated (Cas) proteins [13,16]. Meanwhile, bacteria react differently in response to phage predation [17]. The diversity of bacterial responses is explained by the variety of genetic features of a specific bacteriophage. For example, *Salmonella enterica* engages a SOS response to lytic infection [18], while the response of *Lactobacillus lactis* involves the induction of membrane stress proteins, the d-alanylation of the cell wall, and the maintenance of the proton motive force (PMF) [17,19,20]. Recently, new enzymatic activities in bacteria and archaea, including RNA editing and retron satellite DNA synthesis, were identified as defense mechanisms against phage infection [21].

In its turn, the resistance of phiKZ-like phages to bacterial defense systems based on a double-stranded DNA cleavage has been demonstrated [15,22]. It is intriguing how, in the middle of the phage-infected bacterial cell, an irregularly shaped nucleus-like compartment is formed, which is held in place with a bipolar tubulin spindle [15,23,24]. The shell of the compartment is believed to secure phage genomes from bacterial enzymes that are capable of cleaving phage DNA *in vitro* [15]. Phage proteins associated with DNA replication or transcription are located inside the shell, together with phage DNA [23]. Apparently, the mRNA transcripts are translocated from the pseudo-nucleus to the cytoplasm for phage protein translation by ribosomes, similar to eukaryotic cells.

Despite the apparent importance of this matter, the progress of understanding the maturation of the pseudo-nucleus and of the organization of DNA inside it has mostly been limited to fluorescent studies on live bacterial cells. Recently, structural studies of the infected cell’s cytoplasm (i.e., outside the pseudo-nucleus) were performed, which revealed the newly assembled phage capsids docked to the contiguous shell of the pseudo-nucleus to be filled with the DNA [24]. So far, the spatial organization of the phage DNA inside the pseudo-nucleus shell has eluded the attention of investigators, yet it may shed light on the 3D arrangement of unique nucleus-like compartments.

Here, we used analytical electron microscopy, fluorescent *in situ* hybridization (FISH), real-time PCR, and electron tomography to demonstrate, for the first time, the spatial distribution of phiKZ and bacterial DNA in infected *P. aeruginosa* cells. We have shown that at every moment from the start of the infection, the phage DNA is located inside of different proteinous shell-like structures, which shield it from the impact of bacterial defense systems.

## 2. Materials and Methods

### 2.1. Bacteriophage, Bacterial Strain and Growth Conditions

The strain of *P. aeruginosa* PAO1 and phage phiKZ were generously donated by Dr. V. Krylov (Mechnikov Research Institute of Vaccines and Sera). The PAO1 culture was grown in a LB medium at 37 °C. High-titer phage phiKZ preparations were prepared from lysed infected PAO1 cultures and purified by centrifugation at 10,000× *g* for 10 min. To prepare infected cells for DNA extraction and EM-sample preparation, an overnight PAO1 culture was diluted 1:100 in 1 L of fresh LB medium and, when needed, after reaching OD600 of 0.6, was infected with phiKZ at the multiplicity of infection of 10 (i.e., 10 phages to 1 bacteria cell). In some cases, 100 μg/mL of rifampicin was added to PAO1 5 min before the infection started. Cells were allowed to grow and the infection to spread until indicated time points, and terminated by the addition of 100 μg/mL chloramphenicol and rapid cooling on an ice water bath. For further DNA extraction, cells were harvested by centrifugation (5000× *g* for 10 min), flash-frozen, and stored at −20 °C until use. The efficiency of infection was checked by determining the number of remaining colony-forming units in aliquots of infected cultures collected 5 min post-infection. Only cultures that contained less than 20% of surviving cells were used for further processing.

### 2.2. DNA Extraction, Agarose Electrophoresis, and Real-Time PCR

The DNA was extracted from bacteriophage particles using the standard phenol-chloroform extraction protocol. A GeneJet Genomic DNA Purification kit (TFS, Formerly FEI. Co., Hillsboro, OR, USA) was used to extract the DNA from infected and uninfected PAO1 cells. Equal quantities (400 ng) of total DNA from each sample were first treated with SmaI endonuclease (TFS, Princeton, MA, USA) and then separated in 0.5% agarose gel using a low voltage (2–3 V/cm).

Real-time PCR analysis was performed on a CFX96 Touch Real-Time PCR Detection System (Bio-Rad Laboratories, Irvine, CA, USA) using iTaq Universal SYBR Green Supermix (Bio-Rad Laboratories, CA, USA) according to the manufacturer’s protocol. Pure bacterial and phage DNA in different concentrations (10, 5, 1, and 0.2 ng/μL) were used as standards. Samples of total DNA from infected cells were diluted to 10 ng/μL. Reactions for four standards, and three independent dilutions of each sample were performed simultaneously. The reaction without DNA was performed as negative controls in each series of dilutions. The following primers were used for bacterial and phage genomic DNA:

5′-TCTCTTTCGAGAGGTTGGC-3′ and 5′-TAACCCAGGGCGAGAAGTAC-3′ for a section of the bacterial RpoC gene

5′-GTGTATCATTTAGATAGC-3′ and 5′-GGTCATTGTGAAAGTAC-3′ for the late phage promotor P119L

CFX Maestro Software (Bio-Rad Laboratories, CA, USA) was used for data analysis. The concentration of specific DNA in each reaction was calculated using standards. The resulting concentrations of specific DNAs in three independent dilutions of each sample were normalized against the total DNA concentration of 10 ng/μL, and the average fraction of bacterial and phage DNA for each sample was calculated. To estimate the error, the standard deviation was used.

### 2.3. Fluorescent In Situ Hybridization

Probes for PAO1 and phiKZ genomic DNA were made by digesting genomic DNA with sets of endonucleases (Hin1II, HaeIII, PstI, SphI for PAO1 DNA and Hin1II, HaeIII, HpaII, HindIII, XbaI, NdeI, NheI, NcoI, SphI, BglI for phiKZ DNA) and then labeling digested fragments with Cy5-dCTP using terminal deoxyribonucleotide transferase. PAO1 cells were grown in a LB medium at 37 °C until OD600 = 0.5–0.7, then cells were infected with phiKZ bacteriophage (MOI = 10). 750 ul of cell culture before infection (0 min), after 15 and 30 min of infection were fixed with 4% paraformaldehyde and 0.1% glutaraldehyde for 30 min at room temperature, then centrifuged for 5 min at 2000× *g*, resuspended in PBS, and transferred to flow chamber slides treated with a poly-l-lysine solution. Cells were left to adhere for 10 min at room temperature. Blocking solution (2XSSC buffer, 70% formamide, 1 mg/mL salmon sperm DNA) was applied, and slides were heated at 75 °C for 3 min. Slides were then washed with 70%, 90%, and 96% ethanol for 5 min each at room temperature and dried. Cells were treated with 2xSSC, 50% formamide for 5 min at room temperature, then probes (200 ng/μL) were added in the same buffer. Cells were heated to 94 °C for 3 min, then left at 42 °C for 16 h. Slides were washed with 2xSSC, 50% formamide at 37 °C for 30 min twice, with 2xSSC, 25% formamide at room temperature for 10 min once, then with 2xSSC at room temperature three times, then once again with PBS. Total DNA was stained using DAPI. Micrographs were taken with the Nikon TI eclipse microscope; the Alexa647 channel was used for the Cy5 fluorophore, with exposure of 1000 ms, the DAPI channel was used for DAPI (TFS, MA, USA) staining with an exposure of 5 ms.

### 2.4. Transmission Electron Microscopy

Samples of non-infected cells and phiKZ-infected cells after 5, 10, 15, 20, 30, and 40 min of infection were chemically fixed using glutaraldehyde (2.5%) for 30 min at room temperature. Cells were collected by centrifugation at 5000× *g* at 4 °C. Then, cell pellets were washed twice with sterile PBS, postfixed in 1% osmium tetroxide (Electron Microscopy Sciences, Hatfield, PA, USA) for 30 min at room temperature, and subjected to EMbed 812 Kit (Electron Microscopy Sciences, PA, USA) embedding, according to the manufacturer’s protocol with the replacement of the 100% Propylene Oxide with the 100% Acetone. Thin sections were cut with a diamond knife (Diatome, Nidau, Switzerland) on a ultramicrotome Ultratome III 8800 (LKB, Bromma, Sweden), transferred to nickel grids (400 mesh, Merck, Darmstadt, Germany), covered with collodion, 2% in Amyl Acetate (Electron Microscopy Sciences, PA, USA). Sections were contrasted with gadolinium triacetate (Uranyl Acetate Alternative, TedPella, Redding, CA, USA). Electron microscopy studies were performed using the Libra120 120 kV transmission electron microscope (CarlZeiss, Oberkochen, Germany) at magnification 8000–16,000×.

### 2.5. Sample Preparation for Analytical Electron Microscopy and Electron Tomography

Samples of non-infected cells and phiKZ-infected cells after 15 and 30 min of infection were chemically fixed using a mixture of glutaraldehyde (0.1%) and formaldehyde (2%) for 30 min at room temperature. The cells were collected by centrifugation at 5000× *g* and 4 °C. Then, cell pellets were washed twice with sterile PBS and subjected to LR White (Polyscience, Inc., Warrington, PA, USA) embedding, according to the manufacturer’s protocol. Thin sections were cut with a diamond knife (Diatome) on the ultramicrotomes Ultracut-UCT (Leica Microsystems, Buffalo Grove, IL, USA), transferred to copper 200 mesh grids, covered with formvar (SPI, Washington, DC, USA). Some sections were contrasted with lead citrate.

### 2.6. Electron Tomography

Ultrathin sections were examined with a transmission electron microscope JEM-2100 (Jeol, Tokyo, Japan) with an accelerating voltage of 200 kV. A GIF Quantum ER energy filter with 20 eV energy slit was used to filter out inelastically scattered electrons. Images were recorded with an Ultrascan 1000FTXP CCD camera (Gatan, Pleasanton, CA, USA) at pixel size 0.83 nm. Tomograms were obtained using the SerialEM software [25]. The sample tilt range was set from −60° to +60° with a 2-degree step. Tomograms were reconstructed with the back-projection algorithm in IMOD 4.9. Rendering of the 3D scheme and isosurfaces preparation was accomplished in IMOD 4.9 [26,27].

### 2.7. Energy Dispersive X-ray Spectroscopy (EDX)

X-ray spectra were collected with X-Max 80 mm^2^ EDS detector (Oxford Instruments, Abingdon, UK) in STEM mode and summed over the sample area with a total exposure of over 600 live seconds each.

### 2.8. Electron Energy Loss Spectroscopy (EELS)

Electron energy loss spectroscopy (EELS) spectra and Phosphorus elemental maps were obtained with the Gatan GIF Quantum ER spectrometer (Gatan, CA, USA) in STEM mode. Pixel size was set to 15–20 nm (varies from sample to sample). STEM drift correction was applied after each 40–50 pixels. Each spectrum was obtained at a 6.0 mrad collection angle, 0.25 eV dispersion, and 132 eV energy shift. The spectra from different pixels were aligned to carbon K-edge.

During map processing, the background was fitted with power law over a 115–128 eV energy range, which precedes the phosphorus L2,3 edge, located at 132 eV. Plural scattering effects were corrected using Fourier-ratio deconvolution with the ZLP spectra taken from the same pixel array. The window for Phosphorus signal mapping was set to 132–172 eV.

## 3. Results

### 3.1. Maturation of the Pseudo-Nucleus in P. aeruginosa after phiKZ Infection

To study the changes in the morphology of the pseudo-nuclei over time after their infection, the cells were fixed before and at 5–10–15–20–30–40 min after phage infection and embedded in resin by using the EMbed 812 Kit (Electron Microscopy Sciences, PA, USA), followed by ultrathin sectioning.

**Figure 1:**
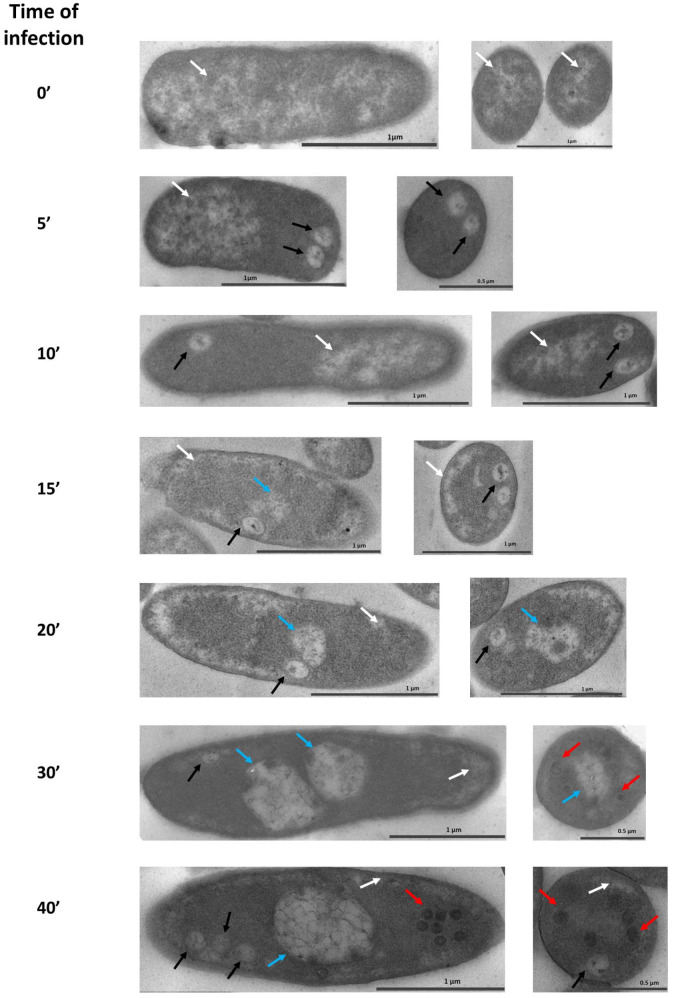
Time-course of the phiKZ infection and pseudo-nucleus maturation in *P. aeruginosa* cells. Left column—minutes after infection. White arrows—bacterial nucleoid; black arrows—round compartments (RC); blue arrows—pseudo-nuclei at different stages of maturation; red arrows—new phage capsids.

**Figure 2:**
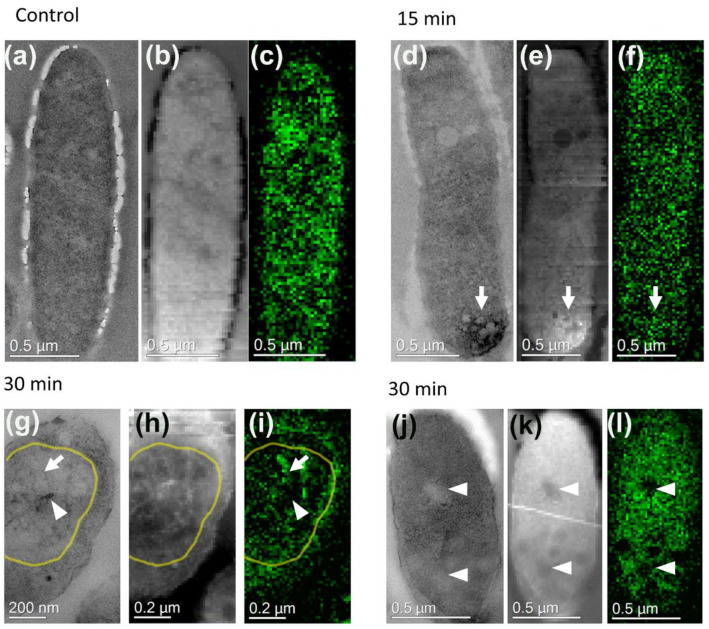
Phage and bacterial DNA distribution in control and infected *P. aeruginosa* cells. TEM image (**a**,**d**,**g**,**j**); HAADF image (**b**,**e**,**h**,**k**), and electron energy loss spectroscopy (EELS) Phosphorus distribution maps through the pseudo-nucleus (**c**,**f**,**i**,**l**). Phosphorous (P) signal was mapped after background subtraction and multiple scattering correction with Fourier-ratio deconvolution. Arrows are pointing to the DNA-containing structures; arrowheads—to the areas, which do not contain the P signal; yellow outlines in (**g**,**h**,**i**) roughly mark the pseudo-nucleus border.

In non-infected cells (0′ line on Figure 1 and Figure 2a,b), the bacterial nucleoid and ribosomes were clearly visible. The nucleoid is diffused inside the cell with a tendency to occupy a central position; no pseudo-nucleus-like structure was detected (Figure 1). The distribution of DNA in the cytoplasm of bacteria was estimated by EELS, according to our previous study [28], using the Phosphorus signal (DNA contains three phosphate groups per each nucleotide) that was detected and mapped onto the cell image (Figure 2). All investigated cells contain the Phosphorus signal, but its spatial distribution differs depending on the post-infection time. In the non-infected cells, the Phosphorus signal was distributed evenly throughout the cytoplasm, reflecting the diffuse position of the nucleoid (Figure 2c).

Upon the addition of phages to the cells, round compartments (RC) ~170 nm in diameter appeared after 5 min of infection close to the cell border. In some cells, more than one compartment was visible (Figure 1, black arrows). We suggested that the number of RC reflects the number of phages that attacked the cell since a high multiplicity of infection was used to decrease the number of uninfected cells: ~10 phage particles per one bacterial cell (Figure S1). These RC are separated from the cytoplasm and contain some electron-dense material (Figure 1), which may be the protein remnants of the phiKZ inner body, subunits of phage RNAPs, or chaperonins [6]. Moreover, after 5 min of infection, we observed the shift of the bacterial nucleoid to the cell pole opposite to the RC (Figure 1), which continued on the 10th min after infection. Up until the 15th min of infection, the bacterial nucleoid moved from the center of the cell to the periphery and occupied a submembrane position. The RCs were kept in the cell, but, at the same time, in some cells we observed a new structure, which became more visible by the 20th min of infection. These structures resembled a mature pseudo-nucleus, albeit smaller and with an underdeveloped internal network (Figure 1, 15′, 20′, blue arrowheads).

**Figure 3:**
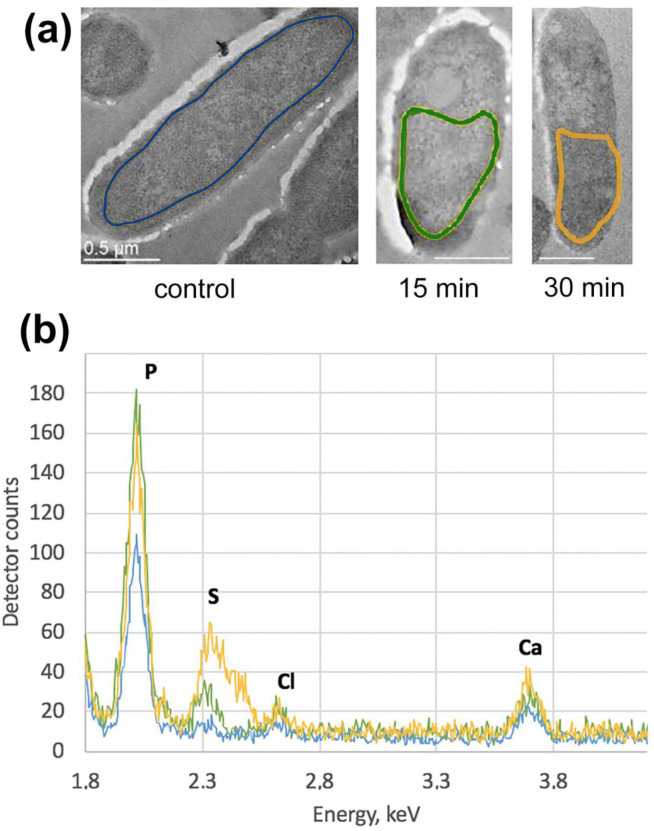
EDX spectra of *P. aeruginosa* control cells, 15 and 30 min after phiKZ infection. (**a**) Colored outlines mark the areas of bacteria subjected for EDX analysis. Bars – 0.5 um.; (**b**) Superimposed EDX spectra from the selected areas, marked in (**a**), normalized to the C peak. Peaks are labeled as follows: P—phosphorus; S—sulfur; Cl—chloride; Ca—calcium.

The even distribution of Phosphorus was detected in the cytoplasm at the 15th min after infection (Figure 2d–f); the overall intensity of the signal was higher compared to control cells. Interestingly, the small sulfur peak appeared on the EDX spectrum (Figure 3). Since the Phosphorous signal had been present in the RC with electron-dense material (Figure 2d–f, arrows), we concluded that these compartments also contain nucleic acid.

After 30 min of infection, the pseudo-nuclei maturated—they became almost spherical and moved closer to the center of the cell (Figure 1, 30′, blue arrowheads), similar to prior observations [15,24]. A single pseudo-nucleus was observed in ~88% of infected cells, while the two-compartment pseudo-nucleus—in ~12% of infected cells, in concordance with previous studies [24]. At the same time, in some cells, the RC remained. The newly synthesized empty and filled phage capsids were detected both close to the surface of the pseudo-nucleus and to the cell wall (Figure 1, red arrows; Figure S1). The average diameter of an empty capsid was 85 nm, a filled one—100 nm, the latter somewhat smaller than the size of a phiKZ mature capsid, which is 145 nm [2,8,29], suggesting that the final maturation of the phage capsids was still ahead. The bacterial nucleoid became less obvious at the periphery of the cell by the 40th min after the start of the infection, which could relate to the degradation of the bacterial DNA. The distribution of the phosphorous signal inside the pseudo-nucleus changes dramatically in cells infected for 30 min (Figure 2i). The bacterial cytoplasm is still filled with an even Phosphorous signal (Figure 2l), but its intensity was higher, comparing to the signal in control cells. Empty new capsids (Figure 2j,k) expectedly did not contain the phosphorus signal (Figure 2l). The height of the above-mentioned sulfur peak increased on the EDX spectrum (Figure 3).

### 3.2. Concentrations of Phage and Bacterial DNA Switch in the Course of phiKZ Infection

To investigate deviations in bacterial and phage DNA concentration in the course of the phage infection, we performed gel-electrophoresis and PCR analysis. Antibiotic rifampicin was added to the cell culture 5 min after phage addition to block the bacterial RNA polymerase, which stop the division of non-infected bacterial cells [30]. It was previously shown that rifampicin does not influence the development of phiKZ infection [3].

**Figure 4:**
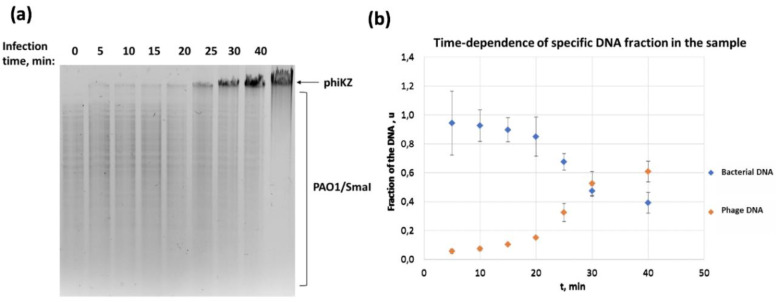
Analysis of the total DNA content from phiKZ-infected cells. (**a**) agarose gel-electrophoresis. Above the gel, the time points are mentioned, where ‘0′ is a non-infected cell, and the last line is the total DNA from phiKZ virions. PAO1/SmaI—bacterial DNA cleaved by SmaI REase. (**b**) real-time PCR results. Blue diamonds-bacterial DNA, orange diamonds-phage DNA.

Total DNA samples were obtained from the non-infected and phiKZ-infected cells at the same time points as those in the morphological studies described above. To qualitatively assess the total DNA samples, agarose gel-electrophoresis was performed with preliminary cleavage of DNA by SmaI REase, which cuts the bacterial genome into small fragments (less than 24 kbp each), but lacks the specific recognition sites in phage DNA (Figure 4a).

In the course of infection, the amount of phage DNA rapidly increased starting at 20 min after infection, while the bacterial DNA did not disappear completely and was still present at least up until the 40th min of infection. To refine these results, we conducted experiments to assess the ratio of phage/bacterial DNA at different time points after infection by real-time PCR. The plot in Figure 4b shows an increase in the fraction of the bacteriophage genome and a decrease in the fraction of the bacterial genome, which started after ~20 min of infection. Since the amount of total DNA for each point was the same, the observed effect is most likely associated with the onset of active replication of phage DNA, starting at the 20th min of infection. This was consistent with our gel electrophoresis results (Figure 4a). However, it is difficult to draw a direct conclusion about what happened to the bacterial DNA. To clarify this, we carried out an additional comparison of the theoretical and experimental comparison of the genomic phage DNA and the bacterial DNA in total DNA samples from cells at different time points after infection. As an internal control of the adequacy of our calculations and simplifications, we analyzed the mass ratio of the phage and bacterium DNA immediately after phage adsorption to the cell. To initiate the infection, at least one copy of the phage genome should enter the cell. At the onset of the infection, the mass ratio of phage DNA to bacterial DNA could be estimated as

(1) $$\frac{MW_{phiKZ}\cdot x}{MW_{PAO1}}$$

where MW_phiKZ_ and MW_PAO1_ are the molecular masses of the bacteriophage and bacterial genome, respectively, and x is the fraction of infected cells in the culture.

The comparison of the estimated values to the experimentally obtained values for two repetitions of the experiment (Table 1) revealed that the experimental values excessed the estimated ones by 1.7–2.5×, which could be explained by the fact that, due to a 10-fold excess of phage particles over the number of cells in the culture, one cell can be attacked by more than one bacteriophage at a time, which is clearly seen on micrographs (Figure 1, 5′ and 10′; Figure S1).

Up until the end of the infection cycle, about 100 new phiKZ phage particles are formed in each infected cell [7], which means that, at least by the end of the infection, there should be ~100 copies of the phage genome. We have shown that in the presence of rifampicin, which blocks further cell division in uninfected cells, the amount of bacterial DNA increased by 1.76 times in 40 min (Table S1). If we assume that the bacterial DNA does not undergo degradation during infection and that the development of the bacteriophage does not prevent the completion of the bacterial genome replication, then the DNAs mass ratio in the sample of the total DNA from the infected cell culture can theoretically be calculated using the formula:

(2) $$\frac{MW_{phiKZ}\cdot x\cdot 100}{MW_{PAO1}\cdot 1.76}$$

If we assume that the DNA of the bacterium in the infected cells is completely destroyed, then we will get another formula for calculating the DNAs ratio:

(3) $$\frac{MW_{phiKZ}\cdot x\cdot 100}{MW_{PAO1}\cdot \left(1-x\right)\cdot 1.76}$$

Thus, we got two boundary values, which we compared with the experimental data (Table 2). The experimental values were 1.3–1.5 times less than the lower boundary of our assessment of the complete absence of bacterial DNA degradation. This means that the bacterial DNA does not undergo complete degradation in the case of infection of PAO1 cells with the phiKZ bacteriophage.

### 3.3. Localization of Bacterial and Phage DNA During Infection

**Figure 5:**
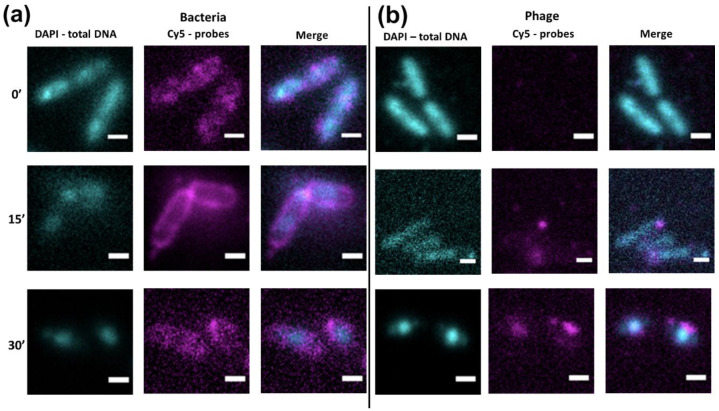
Analysis of bacterial and phage DNA at different times before and after infection of *P. aeruginosa* cells by the phiKZ phage. Results of fluorescent *in situ* hybridization (FISH) (**a**) with bacterial Cy5-probes, (**b**) with phage Cy5-probes. DAPI signal—cyan color, Cy5 signal is colored magenta. Bar—1 um. Time points are indicated on the left.

To determine the intracellular localization of phage and bacterial DNA on the 15th and 30th min after infection, we used FISH and specific Cy5-probes for bacterial (Figure 5a) and phage DNA (Figure 5b).

Non-infected cells were used as a control; Figure 5a demonstrates that in control cells, the signal from the bacterial probes was diffusely spread, while phage probes did not hybridize with control cells. On the 15th min of infection, the signal for bacterial DNA shifted close to the cell membrane, corresponding with TEM data. The signal for phage DNA revealed a condensed state, which may reflect RCs (bright magenta dots on Figure 5b) or early stages of pseudo-nucleus development (condensed signals from DAPI on Figure 5a). Until the 30th min of infection, the phage DNA was localized in the center of the cell as a bright spherical spot reflecting the position of the pseudo-nucleus (Figure 5b). At the same time, the bacterial DNA was diffusely spread within the cytoplasm.

### 3.4. 3D Structure of the Pseudo-Nucleus

**Figure 6:**
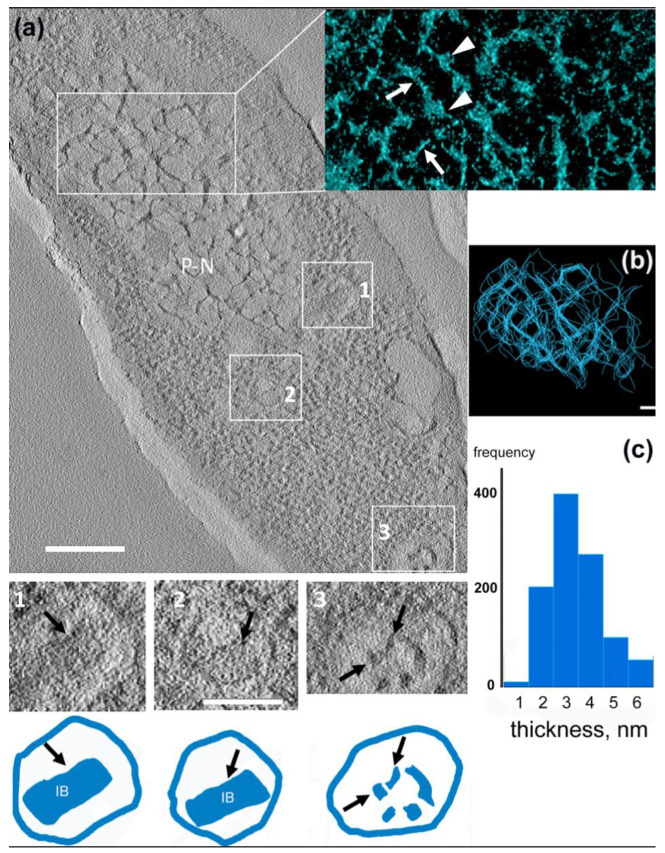
Electron tomography of a phiKZ infected *P. aeruginosa* cell. (**a**) A central slice through the tomogram of an infected cell (30 min after infection). Bar—200 nm. P-N, pseudo-nucleus. Insert—3D representation of the P-N network. White arrows are pointing to DNA strands, arrowheads—to phage DNA-binding proteins. The phage capsids and corresponding gallery images are labeled with rectangles one and two. Bar size—100 nm. Below each gallery image is the matching schematic of the capsid with the inner body (IB) marked with a black arrow; rectangle three and the corresponding gallery image represent a RC. The electron-dense material is clearly visible inside (black arrows). (**b**) The subtomogram representation of the pseudo-nucleus network. Each blue strand represents one DNA helix. Bar—50 nm. (**c**) The distribution (in %) of the average DNA strand’s thickness in the pseudo-nucleus network.

We used electron tomography to reveal the 3D structure of the DNA network inside the pseudo-nucleus (Figure 6a,b). The infected cell at 30 min of infection possesses a mature pseudo-nucleus (Figure 6a); we also detected some partially empty phage capsids (labeled 1 and 2) located close to the pseudo-nucleus border, and the RC (labeled 3)—close to the cell wall. The model obtained in IMOD [26,27] revealed about 90% of the network to be built by thin strands 2 to 4 nm thick (Figure 6c), cross-linked with globular domains of ~10 nm of size. Considering that double-stranded DNA is ~2 nm thick [31], each filament, thus, consisted of ~1–2 DNA double-helical strands (Figure 6b,c). Thereby, the spatial structure of the pseudo-nucleus network (Figure 6b) has been revealed.

Several newly formed capsids were visible close to the pseudo-nucleus border (two are marked in Figure 6a by rectangles one and two), which reveal icosahedral shapes with electron-dense cylinders inside (schematics #1 and #2). We think that these cylinders are the pre-formed inner bodies, which are a characteristic attribute of giant phages [8]. The average dimensions of these cylinders were about 40 nm × 90 nm, which corresponds well with the dimension of the phiKZ’s inner body [8,32].

## 4. Discussion

It was recently shown that phiKZ-like bacteriophages are resistant to many immune mechanisms of bacteria that normally target DNA in vivo, including two subtypes of CRISPR–Cas3, Cas9, Cas12a, and restriction enzymes, such as HsdRMS and EcoRI [15]. These phenomena were excessively studied and linked to the appearance of a large spherical compartment during phage infection in the bacterial cell. The formation of the spherical compartment was revealed using fluorescent microscopy [24,33] and electron tomography [24]. This compartment consists of a protein shell with phage DNA on the inside. Some proteins that participate in the transcription and replication of the phage genome were also found inside the shell. Thus, the compartment resembles the nucleus of a eukaryotic cell in its shape and localization. Moreover, it was suggested that the walls of this compartment shield the phage DNA from bacterial restriction enzymes and CRISPR nucleases. Here, we decided to focus on the fine structure of this pseudo-nucleus compartment and the DNA distribution inside and outside of it. We used transmission analytical electron microscopy and electron tomography to visualize the formation and maturation of the pseudo-nucleus structure in the *P. aeruginosa* cells subjected to the phiKZ infection.

To study the process of the maturation of the pseudo-nucleus compartment, we compared the size, location, and contents of the compartments (Figure 1) induced by phiKZ infection in the bacterial cell, as well as the DNA’s concentration and localization (Figure 4). 5 min after infection, intracellular RCs appeared close to the cell wall (Figure 1, 10’). Sometimes we observed two or more (Figure S1) RCs, which probably indicates a simultaneous attack of the cell by several phages due to the high multiplicity of the infection. The RCs were clearly separated from the cytoplasm, which suggests that they possess a shell. Some electron-dense material (Figure 1) was visible inside these RCs, which is, likely, the protein remnants from the phiKZ virions. Since the phiKZ cell infection is resistant to bacterial defense systems on each stage [15], and the pseudo-nucleus did not form immediately, it can be hypothesized that the protection of the phage DNA at the beginning of the infection is carried out by the RC shell. Somewhat similar structures were observed in cells infected by the giant phage SPN3US from the 5th min of infection [34], which were suggested to be phage proheads maturing within the course of infection. However, the transcription profile for the phiKZ phage was determined earlier [3], and no virion protein genes transcribed at the 5th min of the phiKZ infection were found, so we think that the RCs that were observed here mark the phage’s entrance. Thus, the function and the mechanism of RC formation by giant phages need further research.

Within 15 min after infection, the variety of the intracellular compartment structures and shapes increased: in some cases, they resembled a pseudo-nucleus with a less developed network inside (Figure 1, 15′, arrows), which reflects the graduate maturation of the pseudo-nucleus. A less developed internal network inside the 15th min non-mature pseudo-nucleus may reflect the active stage of phage DNA replication, which starts only by the 20th min of infection, according to the results of our electrophoretic analysis of total DNA preparations and RT-PCR (Figure 4a). The diversity in the shapes of the compartments may stem from the slightly different infection start points in each cell or from the cut planes that are passing through different parts of the cell. In the mature pseudo-nucleus, at the 30th min of infection, a saturated internal network of filaments was clearly visible; the distribution of the branched network clearly corresponded to that of the phosphorous (Figure 2g–i), suggesting that it represents the phage DNA. The empty capsids did not contain the phosphorous signal, proving the lack of DNA (Figure 2j–l).

The genome size of the phiKZ phage is 280 kbp [2]; by the end of the infection, there should be about 100 new phage particles [7], and, therefore, at least 100 copies of the phage genome should be located inside the pseudo-nucleus. Based on the indicated numbers, there should be about 28 Mbp of DNA inside the pseudo-nucleus, which implies the presence of a specific mechanism of phage DNA compactization. Perhaps, some phage proteins inside the pseudo-nucleus represent an analog of the histones of the eukaryotic nucleus, which was consistent with our observations of the globular domains of ~10 nm in size bind to double-stranded DNA (Figure 6a, insert). Future biochemical and molecular biology studies are needed to identify the proteins inside the pseudo-nucleus.

During the process of phage infection, an unexpected distribution of the host nucleoid was revealed. From the 5th min of infection, the nucleoid changed its location inside the cell and shifted itself to cell poles opposite to the phage’s entrance point. Until 15 min after infection, the nucleoid occupied a submembrane position, which was shown by TEM (Figure 1) and FISH (Figure 5) experiments. Later in the infection (30′ and 40′ on Figure 1), the bacterial nucleoid disappeared from the submembrane position; however, according to the FISH results, its remnants remained in the cell until the 30th min of infection. The rearrangement of the *P. aeruginosa* nucleoid at the earlier stages of infection observed here is somewhat similar to the effect of an *Escherichia coli* infection by phage Lambda [35]. In both cases, phage and bacterial DNA were distributed to different poles of the bacterial cell that did not overlap. Since the development programs of the phiKZ and Lambda phages are extremely different, such similarity may reveal one of the general processes in phage infection. On the other hand, the specific mechanism of host and phage DNA separation may be different in each species. Here, we demonstrated that the phage DNA was shielded by numerous shell structures at different stages of the infection process: RCs, pseudo-nucleus, and, later, by the new phage capsid. The mechanism for separating the DNA of the Lambda phage from its host is still awaiting decoding.

It is interesting to note that, after phage infection, the amount of phosphorus that reveals the DNA contents [28] increased in the bacterial cytoplasm, according to EDX data (Figure 3). This may indicate a co-existence of both the phage DNA, whose content increased upon infection, and of the remaining host DNA (Figure 4a). We performed PCR to check this hypothesis, and demonstrated that a considerable level of bacterial DNA is still present even 40 min after phage infection (Figure 4b). These results are in contradiction with those presented by [33], for the related phage 201phi2-1 that infects *Pseudomonas chlororaphis*. Using FISH and fluorescence microscopy, the authors reported the degradation of the host DNA by the 40th min of infection.

We proposed that the remaining host DNA may degrade slowly and be inaccessible for DNases, because it is bound to the host’s stress proteins, like the DNA-binding protein from starved cells (Dps) [36,37,38], the synthesis of which generally increases in stress conditions. It is known that phage infection can elicit diverse stress responses in bacterial cells [17]. It has been shown to affect the regulation of specific stress proteins like the ones related to osmotic, nutrient, and temperature stresses. For example, it was demonstrated that the folding of capsid proteins (P3 and P5) of coliphage PRD1 depends on proteins GroES and GroEL of *E. coli*, which are also responsible for heat shock protection [39]. It was suggested earlier that the giant phiKZ-like phages might directly change the metabolism of the bacterial cell to obtain subsequent support during phage maturation [40].

To check this hypothesis, we performed EDX analysis (Figure 3a) and detected a pronounced peak of sulfur in the bacterial cytoplasm as early as at the 15th min of infection, while its size increased by the 30th min (Figure 3b). This peak may indicate an increase in the content of sulfur-containing proteins, like the above-mentioned Dps. Dps contains 48 Methionines per 24-mer, which possess enough sulfur to be detected by X-ray spectroscopy [28,41]. The increasing contents of Dps lead to the increased protection of bacterial DNA. Notably, the pseudo-nucleus structure, visualized at the 30th min in *P. aeruginosa*, closely resembled isotropic liquid crystalline DNA packaging (Figure S2), which is known to be the first stage of DNA protection against various stress factors in *E. coli* [28]. In liquid crystalline packaging, the DNA-Dps reduces the accessibility of DNA molecules to various external damaging factors, including irradiation, oxidizing agents, and external nucleases. We suggested that the formation of a pseudo-nucleus is a unique safety mechanism, originally developed in bacteria to protect its nucleoid from stress [38,42,43], which may be utilized in the course of evolution by bacteriophages for their needs. However, this hypothesis needs further investigation.

To summarize our results, we can suggest that the maturation of the pseudo-nucleus is a complex multi-stage process connected to phage DNA replication and condensation. Since the very beginning of the phiKZ infection, the phage DNA is, apparently, located first inside the RCs, then inside the pseudo-nucleus, and, lastly, is transferred to the newly-formed capsids. Each of these compartments efficiently separated the DNA from the host defense systems. We have also shown that the development of the phiKZ infection has a significant effect on the placement and structure of the bacterial nucleoid. Moreover, the unique packaging of the DNA inside the pseudo-nucleus or the preceding RCs in the cytoplasm of the live bacterial cell is the main reason for infection sustainability of the phiKZ bacteriophage. We have also shown that the phage DNA 3D organization inside the pseudo-nucleus resembles, to a certain extent, the liquid crystalline network. The high-resolution structure of this network is a subject for future research.

## Figures and Tables

**Table 1:** Comparison of estimated values of the ratio of the phage DNA mass to bacterial DNA mass at 5 min after the infection starts.

Fraction of Infected Cells in Culture, x	Estimated Value of the DNAs Mass Ratio	Experimental Value of the DNAs Mass Ratio
0.8	0.036	0.062
0.65	0.029	0.073

**Table 2:** Comparison of estimated values of the ration of the phage DNA mass to the bacterial DNA mass at the end of the infection, AU.

Fraction of Infected Cells in Culture, x	Estimated Value of DNAs Mass Ratio without Bacterial DNA Degradation	Estimated Value of DNAs Mass Ratio with Full Bacterial DNA Degradation	Experimental Value of DNAs Mass Ratio
0.8	2.03	10.17	1.55
0.65	1.65	4.72	0.97

## Supplementary Materials

The following materials are available online at https://www.mdpi.com/1999-4915/12/10/1197/s1, Table S1: Calculation of the coefficient reflecting of the increase in the amount of extracted bacterial DNA, during cell incubation with antibiotic rifampicin; Figure S1: The empty phage particles attached to the phiKZ-infected cell. Figure S2: Liquid crystalline DNA compactization in starved E. coli cell.
