# Supplementary material for: Maturation of Pseudo-Nucleus Compartment in P. aeruginosa, Infected with Giant phiKZ Phage
Source: Viruses. 2020 Oct 21;12(10):1197. doi: 10.3390/v12101197 (PMC7589130; doi:10.3390/v12101197)
Supplement: Supplementary file 1 [file viruses-12-01197-s001.pdf]

Table 1S. Calculation of the coefficient reflecting the increase in the amount of extracted bacterial DNA during incubation of cells with the antibiotic rifampicin.

**Amount of extracted bacterial DNA before addition of rifampicin, ug**	**Amount of extracted bacterial DNA after 40 min of incubation with rifampicin, ug**	**Increase in the amount of isolated DNA**
10.8	20.8	1.93
11.4	17.4	1.53
9.8	17.8	1.82
	average	1.76

It was shown that in *E.coli* cells rifampicin stops the initiation of a new replication act, but the already begun replication is completed [39]. To test the effect of rifampicin on cells of the PAO1 strain of *P. aeruginosa*, DNA was isolated three independent times from an equal volume of cells before treatment with rifampicin and after 40 minutes of incubation with it. For each repeat, the ratio of the amounts of DNA was determined, reflecting the degree of increase in bacterial DNA after 40 minutes of the experiment. The average increase was 1.76 and this number was used as the corresponding coefficient in the analysis of real-time PCR data.

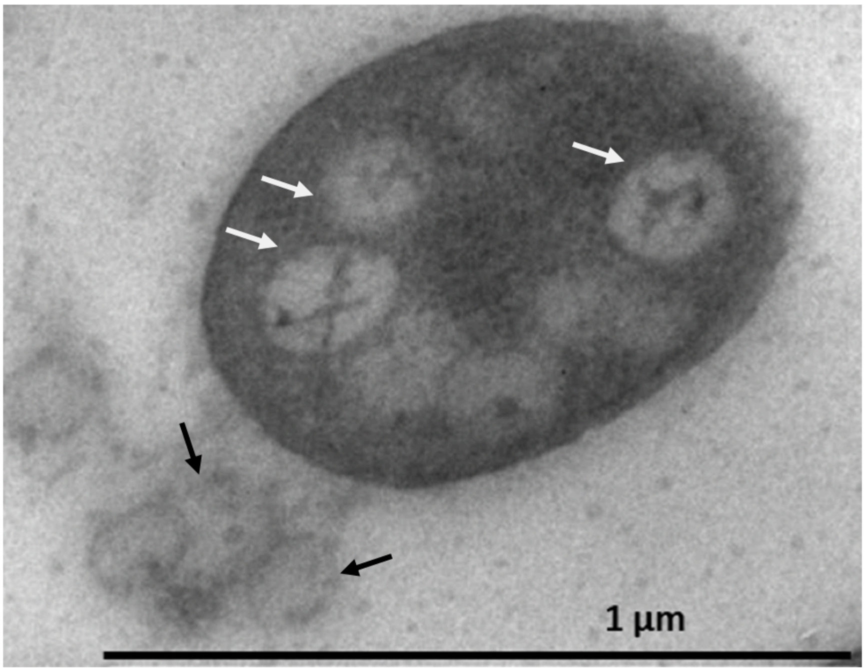

Figure 1S. The empty phage particles attached to phiKZ-infected cell. 10 minutes of infection. White arrows indicate RCs, Black arrows indicate empty phage particles on cell outer membrane.

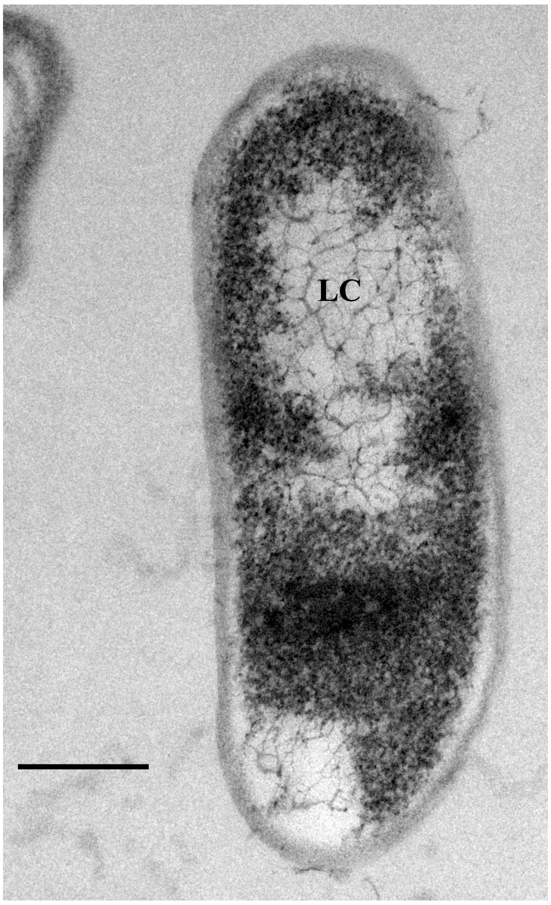

Figure 2S. Liquid crystalline DNA compactization in starved *E. coli* cell. BL21-Gold(DE3)/pET-DPS growing on M9 media with induction of Dps overexpression induced in the linear growth phase, starving 7 months. Bar – 250 nm. LC - Liquid crystalline.
